# Human Chorionic Gonadotropin (hCG) Injections Exacerbating Acute Intermittent Porphyria in a 34-Year-Old Woman

**DOI:** 10.7759/cureus.68651

**Published:** 2024-09-04

**Authors:** Mahesheema Ali, Sahar Iqbal

**Affiliations:** 1 Pathology and Laboratory Medicine, MetroHealth System, Case Western Reserve University School of Medicine, Cleveland, USA; 2 Pathology, MetroHealth Medical Center, Cleveland, USA

**Keywords:** calorie-restricted ketogenic diet, hydroxymethylbilane synthase (hmbs), carbohydrates, human chorionic gonadotrophin hcg, acute intermitent porphyria

## Abstract

A 34-year-old Asian woman arrived at the emergency department (ED) with complaints of sharp, cramping abdominal pain followed by stabbing chest pain that radiated to her back. She also reported numbness, tingling in both hands and feet, and a burning sensation. Upon examination, she exhibited tachycardia and persistently elevated blood pressure. Her lab results revealed low potassium and sodium levels. Despite testing negative for pregnancy, normal hepatic function test, and nerve conduction study, her symptoms persisted, and she was admitted to the intensive care unit for the monitoring and correction of her electrolyte imbalances. She was later discharged but continued to experience symptoms, prompting multiple ED visits. The patient reported the symptoms following a calorie-restricted ketogenic diet and receiving human chorionic gonadotropin (hCG) injections for weight loss, which led the providers to initially diagnose her with "keto flu" due to inadequate nutrient intake. Despite receiving this diagnosis, her symptoms worsened, and she experienced pain throughout her whole body, along with muscle pain and abnormal sensations. Further assessment revealed that her urine was brown and showed abnormal levels of porphyrins, indicating acute intermittent porphyria. A genetic test confirmed the presence of a pathogenic mutation in hydroxymethylbilane synthase (HMBS), leading to a diagnosis of late-onset acute intermittent porphyria.

## Introduction

Porphyrias are a group of disorders that primarily affect the nervous system or the skin, according to the National Organization for Rare Disorders (NORD). They occur when there is an abnormality in the pathway for the production of heme. Porphyrias are distinct clinical syndromes that arise due to a deficiency or defect in a particular enzyme needed for a specific step of the heme synthesis pathway. Although these syndromes have conventionally been classified depending on the predominant system involved (cutaneous vs. neurohepatic), significant overlap occurs, and many porphyrias present with mixed symptoms.

The clinical presentation, severity, and prognosis of porphyrias depend on the deficient enzyme and the corresponding heme precursor or porphyrin accumulation. Acute intermittent porphyria (AIP), the most common acute porphyria, results from the partial absence of the third enzyme in the heme biosynthetic pathway, porphobilinogen deaminase (PBGD), also known as hydroxymethylbilane synthase (HMBS), which affects mainly women [1,2]. The heme pathway intermediates accumulated in various porphyria are neurotoxic or phototoxic and cause associated clinical symptoms with features of acute and intermittent attacks of neurovisceral symptoms that persist for days to weeks, such as abdominal pain, extremities pain with patchy numbness and paresthesia, bulbar paralysis and respiratory impairment, even neuropsychiatric anxiety, and altered consciousness. The frequency and severity of attacks are highly variable and affected by various environmental and other exacerbating factors, such as several medications, hormonal factors, particularly progesterone, ethanol, smoking, starvation or calorie-restricted diet, and stress status [3]. Despite AIP's broad spectrum of clinical symptoms, they are often vague and nonspecific. Diagnosing AIP can be complex due to its varied clinical presentation. To accurately assess prognosis, it is essential to conduct biochemical analysis and molecular genetic tests. AIP is an autosomal dominant disorder with incomplete penetrance. Over 500 disease-causing mutations have been described in the HMBS gene recognized in AIP patients [4]. Herein, we present the case of an adult female patient diagnosed with AIP, harboring a point mutation in the HMBS gene, who initially had multiple visits to the emergency department (ED) due to abdominal pain while on a low-calorie diet.

## Case presentation

A previously healthy 34-year-old woman sought medical attention due to severe abdominal pain, diffuse body aches, and fatigue. She also experienced sudden rapid heart rate and excruciating pain in her chest and arm, along with moderate to severe discomfort. Her vitals showed a blood pressure of 155/110 and a heart rate of 130.

Laboratory tests, including a complete blood count (CBC), electrocardiogram (EKG), and imaging studies of the chest, abdomen, pelvis, and spine, all were unremarkable. The patient started experiencing body pain, severe fatigue, shortness of breath, numbness in the hands and feet, and tingling sensations. She also had a history of diffuse abdominal and muscle pain for five months prior to the acute episode. During a lab investigation, it was found that she had low levels of sodium and potassium in her blood. Further inquiry revealed that she was following a severe calorie-restricted and ketogenic diet (500 calories per day) and taking human chorionic gonadotropin (hCG) injections (one shot in the morning) for weight loss. The patient was diagnosed with the keto flu, and it was recommended that she undergo genetic consultation and further lab evaluation.

Upon further laboratory evaluation, the urine sample showed significantly elevated levels of porphyrins, including δ-ALA, uroporphyrin I, uroporphyrin III, hexacarboxyporphyrin, pentacarboxyporphyrin, coproporphyrin I, coproporphyrin III, and total porphyrins. These findings suggest a potential diagnosis of porphyria. Elevated aminolevulinic acid (ALA) levels are indicative of acute attacks. Additionally, the presence of elevated ketones in the urine suggests that the patient was following a restricted-calorie diet. The patient presented with acute neurovisceral symptoms, but no visible skin symptoms were noted (see Table 1, Table 2, and Table 3 for more details).

**Table 1:** Urine porphyrin laboratory results **creat: creatinine ^a^Outside the reference interval

Urine porphyrin	Reference range	Patient lab values
δ-Aminolevulinic acid (mg/g creat**)	<5.4	34.4^a^
Uroporphyrin I (mg/g creat**)	3.6-21.1	646.1^a^
Uroporphyrin III (mg/g creat**)	≤5.6	342.8^a^
Heptacarboxyporphyrin (mg/g creat**)	≤3.4	38.0^a^
Hexacarboxyporphyrin (mg/g creat**)	≤6.3	15.4^a^
Pentacarboxyporphyrin (mg/g creat**)	≤4.1	61.7^a^
Coproporphyrin I (mg/g creat**)	6.5-33.2	104.5^a^
Coproporphyrin III (mg/g creat**)	4.8-88.6	650.5^a^
Total porphyrins (mg/g creat**)	27.0-153.6	1859.0^a^

**Table 2:** Urinalysis laboratory results

Urinalysis	Reference range and units	Patient's lab values
Color		Yellow
Appearance		Clear
pH	5.0-8.0	6.5
Specific gravity	1.005-1.030	1.015
Protein	Negative	Negative
Blood	Negative	Negative
Bilirubin	Negative	Positive
Urobilinogen	0.2-1.0	1.0
Ketones	Negative	≥160
Leukocyte esterase	Negative	Negative
Nitrite	Negative	Negative
Glucose	Negative	Negative
Pregnancy	Negative	Negative

**Table 3:** Initial laboratory results with corresponding reference intervals L: data is abnormally low; H: data is abnormally high

	Reference range and units	
Sodium, mmol/L	135-148	128 (L)
Potassium, mmol/L	3.3-5.3	3.9
Chloride, mmol/L	97-111	98
Carbon dioxide, mmol/L	21-30	16 (L)
Blood urea nitrogen, mg/dL	8-22	5 (L)
Creatinine, mg/dL	0.50-1.10	0.52
Calcium, mg/dL	8.4-10.4	8.7
Glucose, mg/dL	68-110	112 (H)
Anion gap	10-20	18
Estimated glomerular filtration rate, mL/min/1.73 sqm	≥60	125

By gene sequencing analysis and deletion/duplication testing, the patient was found to have a sequence mutation in the HMBS gene that affects a donor splice site (c.771+2T>C) in intron 11, which confirmed the diagnosis of AIP.

The patient was treated with Panhematin (hemin) therapy. However, the patient's symptoms and porphyrin levels did not improve after treatment. The patient complained of developing brown urine and skin irritation after sun exposure. She was then switched to Givlaari, a small interfering RNA molecule that degrades hepatic delta-aminolevulinic acid synthase (ALAS1) messenger RNA to inhibit the production of the ALAS1 enzyme and thereby reduce the levels of toxic ALA and porphobilinogen (PBG) compounds accumulating inside cells. The patient was advised to discontinue hCG injections and follow a low-calorie diet. The patient's condition improved, and urine δ-ALA and porphyrin levels gradually decreased to normal.

The patient exhibited symptoms of severe abdominal pain, which are present in 90% of acute attacks, as well as constipation, hypertension, tachycardia, hyponatremia, muscle weakness, and dark urine. The next step was to consider lab evaluation. The urine porphyrin lab results indicate AIP, and the gene sequencing analysis confirms the AIP variant. The lab results are crucial for diagnosing AIP, as none of the clinical features are distinct enough to diagnose it based on clinical grounds alone.

## Discussion

Porphyrias are clinically categorized into two primary groups: acute porphyrias which involve sudden neurovisceral attacks and non-acute porphyrias. Acute attacks are associated with the accumulation of the heme pathway intermediates that include porphyrin precursors ALA and consequently PBG from the induced activity of hepatitis ALAS1 and partial hepatic heme deficiency, often in response to the induction of hepatic cytochrome P450 by drugs and other factors. Porphyrias also classified as hepatic porphyrias are AIP, variegate porphyria (VP), hereditary coproporphyria (HCP), and porphyria cutanea tarda (PCT). Erythropoietic porphyrias are congenital erythropoietic porphyria (CEP) and erythropoietic protoporphyria (EPP).

Acute porphyria

Acute porphyrias include ALA dehydratase deficiency porphyria (ADP), AIP, VP, and HCP. All acute porphyrias are inherited in an autosomal dominant manner, except for ADP, an autosomal recessive condition. AIP, VP, and HCP can cause life-threatening neurovisceral attacks.

Acute attacks typically affect females between the ages of 15 and 40 but are rare before puberty. Clinical features of an acute neurovisceral attack may include abdominal pain, vomiting, nausea, constipation/diarrhea, psychologic symptoms, hyponatremia (<135 nmol/L), tachycardia (>80/min), hypertension, motor neuropathy, acute encephalopathy, hemi-/tetraparesis, respiratory paralysis, and sensory neuropathy. The frequency of acute porphyria attacks is highly variable and partly influenced by external exacerbating factors. Some factors known to precipitate the attacks include medications (such as barbiturates, sulfonamides, progestogens, and some anticonvulsants), alcohol, menstrual cycle, restricted calorie intake, infection, and stress [5]. Skin lesions like PCT are present in 80% of VP and less common in HCP. Long-term complications include renal dysfunction, hypertension, and primary hepatocellular carcinoma.

Non-acute porphyria

Some non-acute porphyrias are PCT, CEP, EPP, and X-linked EPP. PCT is the most common form of chronic blistering cutaneous porphyria. CEP is the least common but most severe of the cutaneous porphyrias. The symptoms can range in severity from fetal death in utero to severe skin lesions and anemia in infancy to mild skin lesions similar to PCT that may not appear until adulthood. EPP causes lifelong acute photosensitivity due to a buildup of protoporphyrin IX in the skin. Symptoms typically begin in early childhood, with the average age of onset being one year old. 

Lab evaluation can be performed on urine, blood, and stool specimens to diagnose porphyrias. Testing spot urine PBG and urine creatine is the first-line test for acute porphyria with neurovisceral symptoms. It is recommended to include testing for urine ALA to detect ADP. Even though ADP is exceedingly rare, it can be missed if only the PBG level is tested and comes back normal. Additionally, testing for fractionated urine, fecal, and plasma porphyrins can help detect and differentiate HCP and VP and guide further testing. Isolated porphyrin elevation is not specific for porphyria and can occur in other conditions [6]. In this patient, the urine sample showed significantly elevated levels of porphyrins, including δ-ALA, uroporphyrin I, uroporphyrin III, hexacarboxyporphyrin, pentacarboxyporphyrin, coproporphyrin I, coproporphyrin III, and total porphyrins.

The patient was on a severely calorie-restricted ketogenic diet, involving a 500-calorie intake and daily hCG shots for weight loss, as indicated by elevated ketones in urine. Previous studies have concluded that hCG injections in the luteal phase stimulate estrogen and progesterone production, with progesterone increasing before estrogen but with the increased estrogen levels being maintained for longer periods of time. It is well known that progesterone and low-calorie diet can precipitate AIP attacks. Progesterone and other steroids can prompt the liver to produce ALAS1, the first enzyme in the heme biosynthesis pathway. This can lead to the accumulation of porphyrin precursors when the pathway is deficient, as in AIP [7-9].

More than 500 HMBS gene mutations have been recognized in AIP patients, according to the Human Gene Mutation Database (HGMD) [10]. The mutation identified in this case, HMBS (c.771+2T>C) in intron 11, is an autosomal dominant AIP. This mutation is expected to disrupt RNA splicing, resulting in an absence or disrupted protein product. This is a novel genetic variant of AIP and is not present in the population database yet (ExAC no frequency). Only two AIP cases have been observed in Poland and Italy due to splicing defects and deletions [11,12]. The onset of AIP typically occurs in adulthood, though penetrance is low, and many individuals with biochemical signs of AIP never display clinical symptoms. Identification of a pathogenic mutation not only confirms the diagnosis of AIP but, most importantly, enables the accurate identification of asymptomatic carriers in the AIP family to avoid precipitating factors of the disease and prevent life-threatening acute attacks.

A typical treatment for porphyria involves the administration of hemin therapy or Givlaari and carbohydrates such as glucose to abate acute attacks that downregulate ALAS1. Appropriate recommendations for supportive and symptomatic care and correcting the exacerbating factors are also provided.

## Conclusions

The patient experienced severe abdominal pain while adhering to a calorie-restricted diet. Laboratory tests played a vital role in diagnosing the condition, particularly in distinguishing between acute and non-acute porphyrias, and identifying other contributing factors. It was determined that the patient developed AIP due to exacerbating factors, such as receiving hCG injections for weight loss, which she subsequently had to discontinue. Timely identification of porphyria is essential for initiating appropriate treatment. Diagnosis of acute porphyria should be followed by an investigation of the patient's family members to identify any affected, often asymptomatic, relatives. This allows for advising them to avoid drugs and other factors known to trigger potentially fatal acute attacks.
